# Resequencing the complete *SNCA* locus in Indian patients with Parkinson’s disease

**DOI:** 10.1038/s41531-024-00676-4

**Published:** 2024-04-15

**Authors:** Asha Kishore, Marc Sturm, Kanchana Soman Pillai, Christopher Hakkaart, Divya Kalikavil Puthanveedu, Madhusoodanan Urulangodi, Syam Krishnan, Ashwin Ashok Kumar Sreelatha, Roopa Rajan, Pramod Kumar Pal, Ravi Yadav, Gangadhara Sarma, Nicolas Casadei, Thomas Gasser, Peter Bauer, Olaf Riess, Manu Sharma

**Affiliations:** 1 Comprehensive Care Centre for Movement Disorders, Sree Chitra Tirunal Institute for Medical Sciences and Technology, Kochi, Kerala India; 2 Parkinson and Movement Disorder Centre, Centre for Excellence in Neurosciences, Aster Medcity, Kochi, Kerala India; 3 Institute for Medical Genetics and Applied Genomics, University of Tübingen, Tübingen, Germany; 4 Centre for Genetic Epidemiology, Institute for Clinical Epidemiology and Applied Biometry, University of Tübingen, Tübingen, Germany; 5 Department of Neurology, All India Institute for Medical Sciences, New Delhi, India; 6 Department of Neurology, National Institute of Mental Health and Neurosciences, Bengaluru, Karnataka India; 7 Department for Neurodegenerative Diseases, Hertie Institute for Clinical Brain Research, University of Tübingen, Tübingen, Germany; 8 German Center for Neurodegenerative Diseases (DZNE), Tübingen, Germany; 9 Centogene GmbH, Rostock, Germany; 10 University Medicine Rostock, Internal Medicine III, Hematology, Rostock, Germany

**Keywords:** Parkinson's disease, Neurodegeneration

## Abstract

The genetic loci implicated in familial Parkinson’s disease (PD) have limited generalizability to the Indian PD population. We tested mutations and the frequency of known mutations in the *SNCA* gene in a PD cohort from India. We selected 298 PD cases and 301 age-matched controls for targeted resequencing (before QC), along with 363 PD genomes of Indian ancestry and 1029 publicly available whole genomes from India as healthy controls (IndiGenomes), to determine the frequency of monogenic *SNCA* mutations. The raw sequence reads were analyzed using an in-house analysis pipeline, allowing the detection of small variants and structural variants using Manta. The in-depth analysis of the *SNCA* locus did not identify missense or structural variants, including previously identified *SNCA* mutations, in the Indian population. The familial forms of *SNCA* gene variants do not play a major role in the Indian PD population and this warrants further research in the under-represented population.

## Introduction

The global burden of Parkinson’s disease (PD) was estimated in 2020 to be 9.4 million and about 10% of these patients live in India^1,2^. However, they were underrepresented in large-scale genetic studies conducted so far^3^. Most genetic loci implicated in familial PD were identified in the European and East-Asian populations and many of these mutations were seldom seen in Indian PD patients, including the rather frequent *G2019S* variant in the *LRRK2* gene^4^. The discovery of PD caused by *SNCA* mutations and the presence of alpha-synuclein within Lewy bodies, the pathological hallmark of PD, triggered great interest in the pathogenic role of the *SNCA-*encoded protein in PD^5^. The *p.Ala30Pro*, *p.Glu46Lys*, *p.His50Gln*, *p.Gly51Asp*, *p.Ala53Thr*, p.Ala53Glu, *p.Ala53Val*, and most recently the *p.Ala30Gly* are the major *SNCA* mutations discovered so far^6–14^. Duplications and triplications of the *SNCA* locus were also reported causing PD^15–17^. However, reports of *SNCA* mutations from the Asian population are exceedingly rare^18^. In the present study, we re-sequenced the entire *SNCA* locus and retrieved locus-specific genetic information from an ongoing Indian PD genome sequencing study to look for known, as well as unknown mutations and/or structural variants, in a large cohort of Indian PD patients and healthy controls.

## Results

### Targeted resequencing and whole genome sequencing

**Fig. 1:**
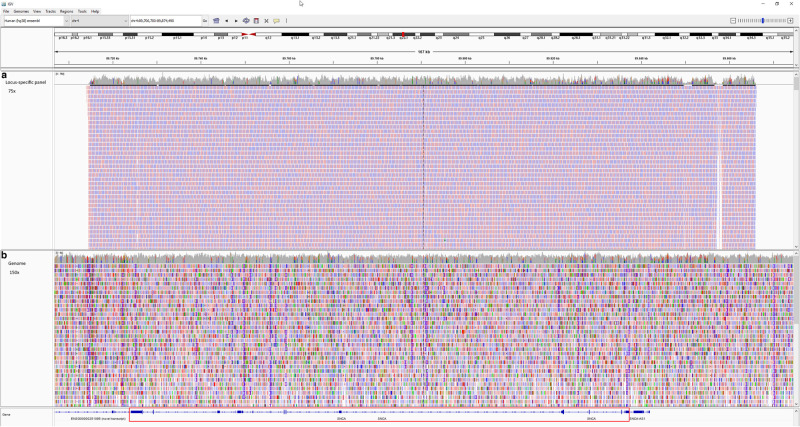
In-depth sequencing analysis of an SNCA locus. The integrative genomics viewer (IGV) panel displaying the coverage of *SNCA* locus in targeted resequencing (upper panel; 1**a**) and whole-genome (lower panel; 1**b**).

For the *SNCA* locus targeted resequencing cohort, the mean sequencing depth was 244x. On average, 97.85% of the 151-kbp target region was covered with at least 20× depth (Fig. 1a). For the whole-genome cohort, the mean sequencing depth was 29.6×. On average, 94.70% genome was covered with at least 20× depth (Fig. 1b). Of the 599 samples (298 cases and 301 controls), ten samples were of low quality (average target region read depth of less than 50× or more than 93% of the target region covered by less than 20×). Further, three samples that were duplicated, were excluded from further analysis. Thus, a total of 288 PD cases and 298 controls were available for the variant analysis from the targeted resequencing cohort, while 363 genomes were used for whole-genome sequencing. Taken together, in a combined total of 651 cases and 1327 controls from India, we did not identify previously described mutations and/or missense and/or structural variants of the *SNCA* gene in the Indian cohort.

## Discussion

The Indian population because of its unique genetic makeup (due to widespread founder events) led to the accumulation of population-specific genetic variants^19^. Thus, the cataloging of disease-specific variants will pave the way for precision medicine in India. This is the largest study in which an in-depth analysis of the *SNCA* locus was performed by employing targeted resequencing as well as retrieving WGS data from our ongoing study and population-specific control genomes from India to detect unknown variants, including structural variants and assessing the prevalence of the previously identified all major *SNCA* mutations in the Indian population. The comprehensive analysis excluded the role of the rare forms of the previously identified major monogenic *SNCA*-dependent PD mutations in the Indian population (Table 1).

**Table 1:** Reported *SNCA* missense mutations in familial and sporadic Parkinson’s disease among various ethnicities

*SNCA* mutation	Region of origin	Ethnicity	Familial/sporadic PD	Reference
*p.Ala53Thr*	Italian, Greek, Finnish	European	familial	^6,9,33^
*p.Glu46Lys*	Spanish	European	familial	^7^
*p.Ala30Pro*	German	European	familial	^8^
*p.Ala30Gly*	Greek	European	familial	^10^
*p.Thr72Met*	Turkish	Turkish	familial	^23^
*p.His50Gln*	English–Welsh, English	European	familial, sporadic	^11,12^
*p.G51D*	French	European	familial	^14^
*p.M5T*	Chinese	Chinese	familial	^18^
*p.Val15Asp*	Chinese	Chinese	sporadic	^25^
*p.Met127Ile*	Chinese	Chinese	sporadic	^25^
*p.A53V*	Chinese	Chinese	sporadic	^15^
*p.P117S*	Chinese	Chinese	sporadic	^15^
*p.A18T*	Polish	European	sporadic	^26^
*p.A29S*	Polish	European	sporadic	^26^

The first pathogenic mutation in the *SNCA* gene (*p.Ala53Thr*) was reported in 1997 in an Italian kindred and three unrelated families of Greek origin^6^. Later, mutations derived from a common founder were described in Finnish (*p.Ala53Glu*) and 3 Greek families (*p.Ala30Gly*)^9,10^. Further, duplications and triplications of the *SNCA* locus which correlated with disease severity, were reported to cause familial parkinsonism^20–22^. After the discovery of the *p.Ala*53*Thr* mutation, *p.Ala*30*Pro* and *p.Glu*46*Lys* mutations were identified as single families, of German and Spanish origin, respectively^7,8^. Another mutation, *p*.*Thr*72*Met*, was recently found in four members of two Turkish families^23^. Finally, the *p.His50Gln* mutation was identified in a Canadian patient of English–Welsh ancestry with PD and a positive family history of parkinsonism and dementia, and in a single English patient with sporadic, pathologically-confirmed PD^11,12^.

There are scarce reports of *SNCA* mutations from Asian populations. A novel autosomal dominant inherited *p.Met5Thr* mutation was found in a recent Chinese study of 155 PD patients^18^. Though single nucleotide polymorphisms in the *SNCA* gene are known to increase the risk of sporadic PD, none of the initially identified pathogenic substitutions were found to be involved^24^. A novel variant, *p.Val*15*Asp*, and another unclear variant, *p.Met*127*Ile*, in the *SNCA* gene were found in a Chinese study of 191 sporadic PD and 200 controls^25^. Further, two likely pathogenic mutations, *p.Ala53Val*, and *p.Pro*117*Ser* were found among 3 Chinese patients in a study of 433 sporadic PD cases and 543 age-matched controls^15^. Two novel substitutions, *p.Ala18Thr*, and *p.Ala*29*Ser*, were each found in a single patient with sporadic late-onset PD in a Polish study of 629 PD patients^26^.

Three previous studies from India failed to detect known mutations in the *SNCA* gene in their PD cases. These include a study with 140 PD patients and 201 normal controls that were tested for the *p.Ala53Thr*, *p.Ala30Pro*, *p.Glu46Lys* mutations^27^, and another for the *p.Gly88Cys* or *p.Gly209Ala* mutations in 169 patients, respectively^28^. A third study which re-sequenced 6 exons of the *SNCA* gene in 100 PD patients and ethnically-matched controls, also revealed no mutations in the gene in this population^29^.

Based on these reports from both south and north-Indian PD cases which tested known mutations, and the resequencing and WGS data from the entire *SNCA* locus in the South Indian and pan-Indian PD cases respectively in the current study, it is evident that known *SNCA* mutations have no major role in PD in the Indian PD population. In contrast to the previously published studies from India, we re-sequenced the complete locus to identify any potential structural variants in our cohort that could have been missed in the previously published studies. The present study screened a larger cohort to assess the role of *SNCA*-monogenic mutations in PD patients in the Indian population. Currently, a pan-Indian study to decipher the role of common genetic variability of PD, including the role of *SNCA* variants, is underway in the Indian population^30^.

The current information on the genetic susceptibility to PD worldwide relies mostly on data from European, North- American, and East- Asian populations. To our knowledge, this is the largest study analyzing the entire coding region of the *SNCA* gene in a cohort of Indian patients with PD which revealed an important finding that the rare variants in the *SNCA* gene responsible for monogenic-PD in other populations cannot be implicated in the Indian PD population.

## Methods

### Targeted resequencing cohort

A total of 298 cases and 301 age-matched healthy controls of Indian ancestry were included in the study. All PD cases were diagnosed at the Movement Disorder clinic of a tertiary-care university hospital (Sree Chitra Tirunal Institute for Medical Sciences and Technology-SCTIMST) in Kerala, South India. All patients were diagnosed by a movement disorder specialist using the United Kingdom Parkinson’s Disease Brain bank criteria^31^. Data related to both sporadic and familial PD (*n* = 25) were compiled for research purposes in the data bank of the Movement Disorder clinic. The mean age at onset was 49.9 years (range, 24–80) Ethnically- matched healthy controls, unrelated to patients, were also regularly recruited to build a comprehensive control group for the study. Before inclusion in the study, the controls were examined for any neurological disorders and queried for any family history of neurodegenerative disorders.

### Whole-genome sequencing cohort

From our ongoing whole genome sequencing study, a total of 363 samples were used for analysis. The mean age of onset was 52.15 ± 10.35, and the male-to-female ratio was 2.1:1. All cases were diagnosed by Movement disorder specialists using the same criteria. The institutional ethics committees of all centers approved the study. All participants signed informed consent.

### Targeted resequencing of *SNCA*

5 ml of blood was collected from each volunteer using venipuncture, and genomic DNA was extracted using the salting out method for targeted resequencing as well as for whole-genome analysis^32^. Resequencing was performed at the Core facility of Applied Transcriptomics and Genomics at the Institute of Medical Genetics and Applied Genomics, University Hospital of Tübingen, (Tübingen, Germany). A total of 298 cases and 301 controls were selected for resequencing. The *SNCA* locus resequencing was performed by using several long-range PCRs to amplify the 151 kb SNCA locus. The PCR amplicons were turned into a sequencing library using the “Nextera XT DNA Library Preparation” kit (Illumina, San Diego, CA, USA). Sequencing of the libraries was performed on the NextSeq500 sequencer (Illumina, San Diego, CA, USA) using 75 bp paired-end sequencing. Generated sequences were processed using the open-source pipeline megSAP (https://github.com/imgag/megSAP/tree/2022_08) based on the GRCh38 reference genome.

### Whole-genome sequencing

For DNA sequencing, 350 ng of genomic DNA was fragmented to ~450 bp pairs using the DNA PCR-Free Prep, Tagmentation (Illumina). The resulting libraries typically present a concentration of 1.5–3 ng/µl and are sequenced as paired-end 150 bp reads on an Illumina NovaSeq6000 (Illumina) with a sequencing depth of approximately 120 Gb. Generated sequences were processed using the open-source pipeline megSAP (https://github.com/imgag/megSAP/tree/2022_08) based on the GRCh38 reference genome. *SNCA* locus spanning 151 kb region was selected for screening PD genomes.

### IndiGenomes

A publicly available database was used to search for putative variants in the control genomes ascertained from different regions of India. In brief, a total of 1029 self-declared healthy individuals underwent whole-genome sequencing to develop a comprehensive compendium of genetic variants in the Indian population. For details, please see ref. ^19^.

### Genomic analysis

For targeted resequencing and *SNCA* locus data from whole genomes; the megSAP pipeline was used (https://github.com/imgag/megSAP). In brief, megSAP performs quality control, read mapping, variant detection, as well as comprehensive annotation of variants. Detailed information about tools used by megSAP and tool versions can be found in the megSAP documentation. For the main analysis steps the following tools were used: BWA-mem2 for read mapping (https://github.com/bwa-mem2/bwa-mem2), freebayes for small variant calling (https://github.com/freebayes/freebayes), and Manta for structural variant calling (https://github.com/Illumina/manta). For previously described mutations, we directly search for known mutations in our cohort. To find unknown PD variants, we filtered the detected variants using two main criteria: (1) The variant must be a protein-altering splice region, and (2) the variant should have a maximum allele frequency of 0.01% in gnomAD, including subpopulations.

### Reporting summary

Further information on research design is available in the Nature Portfolio Reporting Summary linked to this article.

### Supplementary information

Reporting Summary

## Data Availability

The data used in this study is under restriction by the ethical committees, and thus cannot be made publicly available. However, the researcher can send a request to the corresponding author for data access. Based on the feasibility of the project, the corresponding author will provide guidelines to the researchers regarding data access to our cloud portal to perform and conduct their study for which any investigator will request our data.

## Code availability

The publicly available analytical tools were used for the analysis. megSAP pipeline (https://github.com/imgag/megSAP), BWA-mem2 (https://github.com/bwa-mem2/bwa-mem2), freebayes (https://github.com/freebayes/freebayes), Manta (https://github.com/Illumina/manta).
